# Reconfigurable Spoof Plasmonic Skyrmion Electronics for Deformation‐Invariant Multimode Sensing

**DOI:** 10.1002/advs.75071

**Published:** 2026-04-08

**Authors:** Zengxiang Wang, Xiangzheng Kong, Xiaojun Huang, Ying Tian, Wang Yao, Xia Xiao, Zhuo Li, Yu Luo

**Affiliations:** ^1^ College of Communication and Information Engineering Xi'an University of Science and Technology Xi'an China; ^2^ State Key Laboratory of Advanced Materials for Intelligent Sensing Tianjin Key Laboratory of Imaging and Sensing Microelectronic Technology School of Microelectronics Tianjin University Tianjin China; ^3^ College of Energy and Mining Engineering Xi'an University of Science and Technology Xi'an China; ^4^ State Key Laboratory of Microwave Photonics School of Electronic and Information Engineering Nanjing University of Aeronautics and Astronautics Nanjing China

**Keywords:** deep‐subwavelength, flexible microwave resonator, plasmonic skyrmions, space‐coiling metasturcture

## Abstract

The advancement of wearable electronics is often hindered by motion artifacts and miniaturization constraints, especially in dynamic physical environments such as skin interfaces. To address these challenges, we introduce a flexible multimode sensor based on reconfigurable spoof localized surface plasmon (SLSP) skyrmions with integrated multi‐capacitor loading. This design achieves tight compression of all equidistant resonant modes, yielding an ultrahigh quality factor of 164.29 within a deep‐subwavelength footprint of only λ/14.5 in diameter. We systematically investigate three distinct topological configurations—circular, square, and heart‐shaped—with identical effective skyrmion lengths, and demonstrate that all multi‐resonant modes retain consistent frequencies regardless of shape, underscoring exceptional topological robustness. Fabricated on a flexible polyimide substrate, the SLSP skyrmions exhibit remarkable bending stability, maintaining stable resonant responses under various deformation conditions, as validated by both simulation and experiment. Furthermore, through capacitance loading, the average normalized sensitivity of multimodal sensing is enhanced from 2.5% to 3.3% when exposed to materials of different dielectric constants. Our work establishes a new paradigm for motion artifact‐free, flexible sensing with ultra‐compact form factors and high quality factor (Q‐factor), highlighting the significant potential of SLSP skyrmions in next‐generation wearable electronic applications, including electronic skin and conformal health monitors.

## Graphical abstract

A flexible multimode sensor based on capacitively loaded spoof plasmonic skyrmions enables deformation‐invariant dielectric sensing with ultra‐compact size and high Q‐factor. Near‐equidistant resonances remain stable under shape change and bending, while capacitor loading improves bending robustness and multimode sensitivity, highlighting strong potential for wearable electronics and conformal health monitoring.

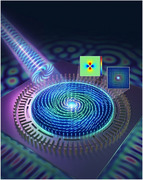

## 1 Introduction

Localized surface plasmons (LSP) are non‐propagating surface waves resulting from the resonance between confined electrons near metallic nanoparticles and incident electromagnetic waves, characterized by high local field enhancement [1]. Initially, they were only applied in the optical field. Spoof localized surface plasmons (SLSP) was proposed to mimic the behavior of LSP and exhibited exciting features such as subwavelength confinement and near‐field enhancement, extending LSPs into the microwave and terahertz fields [2]. Microwave sensors SLSP‐based offer miniaturization, high quality factor (Q‐factor) (high resolution), and high sensitivity, attracting extensive attention from researchers in recent years. The structure design of SLSP‐based sensors is highly flexible, enabling extremely high sensitivity. They can also be integrated with passive or active components for dynamic tuning and are directly compatible with conventional microstrip and coplanar waveguide circuits [3].

Wireless modules, providing telecommunications, sensing and energy harvesting functions through radio frequency (RF) electronics, are essential components of skin‐interfaced stretchable electronics [4, 5, 6, 7, 8, 9, 10, 11, 12]. However, all flexible substrates share the drawback of directly affecting the frequency‐dependent characteristics of almost all RF components. Recent reports on stretchable RF components have shown that even under relatively low elastic strain, substantial changes in electrical performance occur, such as frequency shifts of resonators [13, 14]. These variations significantly reduce wireless signal strength or sensing performance in flexible electronic systems, especially in physically dynamic environments such as the skin surface. Recently, researchers proposed the concept of SLSP skyrmions based on space‐coiling meta‐structure, which supports magnetic SLSP without requiring careful control of external interference in the microwave range [15, 16, 17, 18]. The spectral response of meta‐structure exhibits near‐equidistant resonances that remain unaffected by arbitrary shape deformations, even in the introduction of abrupt angular features and highly asymmetric contours. Furthermore, this approach overcomes the limitation of conventional microwave sensors, which typically operate in narrow bands with only one or a few resonant modes. Capitalizing on these strengths, SLSP skyrmions pave the way for characterizing dielectric responses over a broadband multi‐mode with topological stability and mechanical adaptability, thereby finding potential uses in flexible sensing, electronic skin, and ultra‐compact resonators.

In this paper, we propose a capacitively tuned SLSP skyrmions‐based high‐performance deformable RF flexible resonator with bending invariance for characterizing the dielectric properties of target materials. Fabricated as a space‐coiling meta‐structure, this device demonstrates a nearly equidistant multi‐resonance response, with the fundamental mode resonance diameter compressed to λ/14.5. To stimulate and characterize these resonance responses, the source was fed vertically into the center of skyrmion structure, with the *S* parameters being directly measured. By analyzing the capacitor loading positions and different capacitances, an optimal capacitive tuning strategy is obtained. Due to the deep‐subwavelength scale field compression and strongly localized evanescent fields in all of modes, proposed resonator has an ultra‐compact size and achieves excellent sensing performance across a broadband multi‐frequency range. To ‌prove topological robustness against shape deformation, we fabricated and compared three SLSP skyrmions variants (circular, square, and heart) of equal effective waveguide length. Furthermore, we enhance the bending stability of the sensor by loading capacitive elements. Through analysis of capacitor placement, we achieve a microwave sensor with high Q‐factor, high sensitivity, and high resolution. These works provide a novel pathway for broadband dielectric detection using multi‐resonant modes and facilitate the future implementation of SLSP skyrmions in sensors that require ultra‐compact and topological stability. Different from single‐resonance sensing, tracking multiple near‐equidistant skyrmion modes enables broadband multi‐frequency permittivity sampling on the same ultra‐compact platform, and provides redundancy for cross‐verification and outlier rejection in wearable scenarios where coupling and placement variations are inevitable.

## 2 Theoretical Modeling and Design of SLSP Skyrmions

**FIGURE 1:**
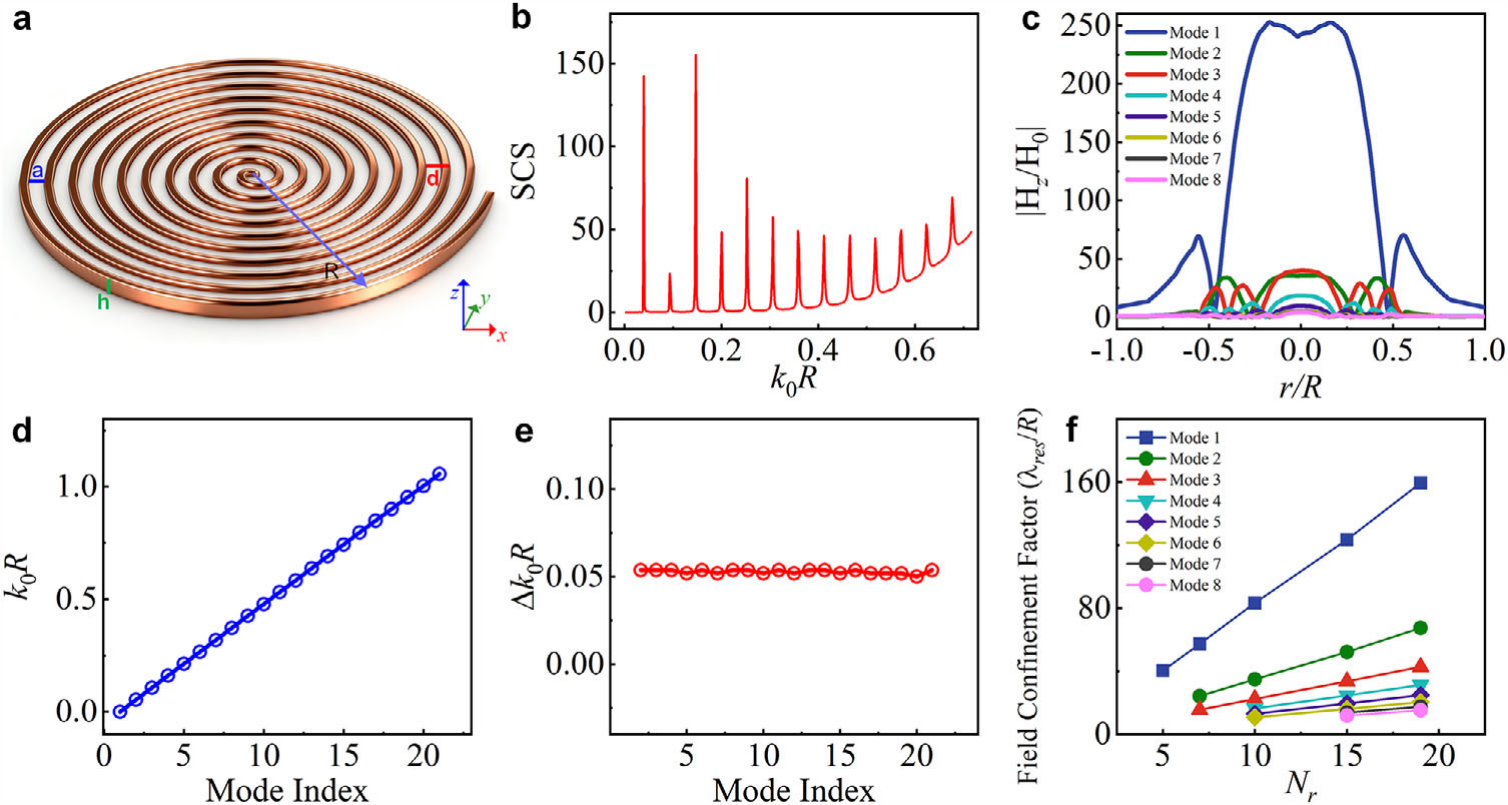
(a) 3D schematic of the spoof localized surface plasmons (SLSP) skyrmions. (b) Scattering cross‐section of a meta‐structure with *N_r_* = 19 and a duty cycle (a/d) of 0.15/0.45 mm, in which Plane‐wave excitation: ***k*** = − y, **E** ∕∕ *x*. (c) Radial profile of the magnetic field enhancement for the resonant modes, normalized to the impinging field. (d) Normalized resonant frequency *k*_0_ *R* of the first 19 modes. (e) Normalized frequency spacing Δ*k*_0_ *R* vs. mode index. Δ*k*_0_ *R* is calculated as the difference between normalized frequencies of adjacent modes. (f) Dependence of the field confinement factor (λ*_res_*/*R*) for the fundamental and higher‐order modes on the *N_r_*.

A metasurface employing a uniaxial spiral metallic strip was implemented, in which the details of the dimensional parameters were shown in Figure 1a. The radius *R* of the spiral structure is defined by the relationship *R* = *N_r_* × *d*, where *N_r_* is the number of spiral turns. Therefore, the continuous spatially coiled air‐gap region confines the electromagnetic field at a deep‐subwavelength scale. The space‐coiling meta‐structure, fashioned from a single, connected groove of a perfect electric conductor, was numerically analyzed. This groove exclusively supports a radially lobed, magnetic SLSP mode, which produces a multi‐resonance spectrum in its scattering cross‐section

(SCS). This spectrum is marked by evenly spaced, needle‐like peaks at a deep sub‐wavelength scale (characteristic size: *R* ∼ λ/430, *N_r_* = 19), as shown in Figure 1b. The spectrum consists of resonances at frequencies defined by *f*_0_ + *n*Δ*f_m_* (*n* = 0, 1, 2, …), with *f*_0_ being the fundamental resonance and Δ*f_m_* the free spectral range (FSR). This creates a comb‐like pattern with successive increments of Δ*f_m_*. These parameters are governed by the *N*_r_. The resulting spectrum is well‐explained by a serpentine waveguide model, wherein the resonance positions and FSR are accurately described by a semi‐infinite effective waveguide model (described in Note S1). At deep subwavelength scales, the contribution of electric modes to the SCS becomes negligible (described in Note S2 and Figure S1), affirming the purely magnetic skyrmions resonance modes. Distinct from classical magnetic Mie resonators that exhibit parallel **E** and **J** fields [19], this design exploits structural anisotropy to support a quasi‐static mode exhibiting orthogonal radial **E** and azimuthal **J** distributions. This peculiar resonance mode facilitates extreme field enhancement (Figure 1c). Figure 1d,e demonstrates a linear relationship between the resonance frequency *k_0_R* and resonance linewidth Δ*k_0_R* with the mode index, evidencing equidistant resonances. Figure 1f reveals a proportional relationship between the *N_r_* and the field confinement factor (resonance wavelength vs. radius, *λ_res_*/*R*) for the fundamental and higher‐order modes, demonstrating that greater compactness is achieved by increasing the number of coil turns.

When designing sensors for characterizing material permittivity, the sensitivity is of primary interest. Sensitivity is defined as Δ*f* induced by a local permittivity change Δ*ε*(*r*, *f*). The Δ*f* resulting from a localized permittivity perturbation Δ*ε*(*r*, *f*) is governed by cavity perturbation theory, which predicts a proportionality to the electric field energy magnitude over the target volume *V* [20]:

(1) $$\Delta f=-f\underset{V}{\;\int \int \int \;}\Delta \varepsilon \left(r,f\right){\left|E(r)\right|}^{2}d^{3}r$$

For the field distribution characteristics, shown in Figure 1c, thus the fields within this deep‐subwavelength SLSP skyrmions resonator are highly confined and significantly enhanced. This strong field‐matter interaction holds considerable potential for introducing substantially larger sensor responses. Furthermore, owing to the characteristic of equidistant multi‐resonance, broadband multi‐frequency sensing can be realized on this ultra‐compact platform without requiring resonator replacement for each resonance. These properties make it an attractive platform for an ultra‐compact plasmonic sensor that operates at multiple frequencies and can characterize material permittivity over a broad bandwidth.

All resonance modes exhibit an axially symmetric magnetic field distribution, characterized by a unit vector that rotates in space by an integer multiple of π radians. The skyrmions number *S*, serves as a quantitative metric for evaluating the topology of the vectorial magnetic fields, and is expressed by [21]

(2) $$S=\frac{1}{4\pi }\;\int \int \;h\cdot \left(\frac{\partial h}{\partial x}\times \frac{\partial h}{\partial y}\right)dxdy$$

**FIGURE 2:**
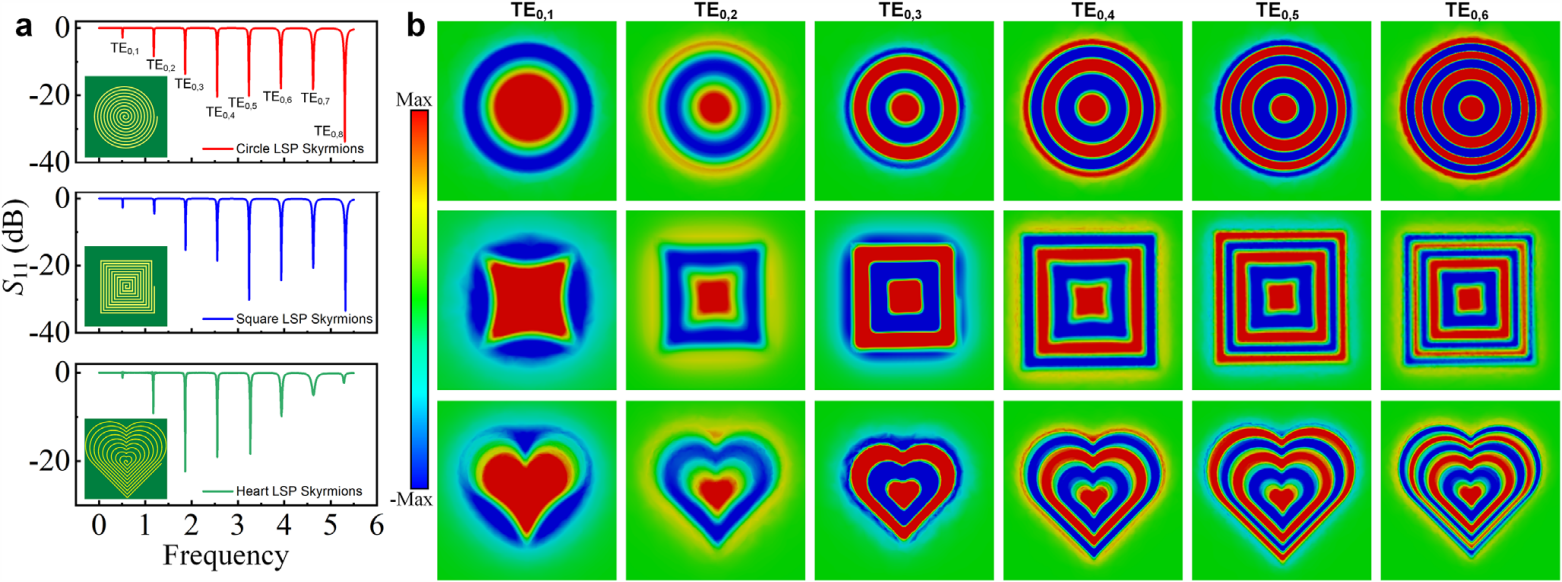
Deformation‐invariant properties of SLSP skyrmions. (a) topological structure and near‐equidistant multi‐resonant response spectra. (b) Magnetic field patterns for the first six modes in three distinct deformed geometries.

Here, h=(Hx,Hy,Hz)/(Hx,Hy,Hz)|H||H| represents the local unit vector of the field, and the integrand $h\cdot (\frac{\partial h}{\partial x}\times \frac{\partial h}{\partial y})$ denotes the skyrmions density. Therefore, *S*, computed from this density, serves as a topological invariant that quantifies the total number of full *π* rotations performed by the magnetic field vectors in the radial direction. The fundamental (*π*‐twist) mode represents a basic skyrmions with a topological charge of 1, while the second (2*π*‐twist) mode constitutes a skyrmions (charge of 0) [22, 23, 24]. Higher‐order modes correspond to multi‐*π*‐twist topological solitons (TSs) [25, 26]. Opposing twists in adjacent regions cancel one another. Therefore, odd‐numbered modes exhibit a net topological charge of 1, while even‐numbered modes have a net charge of 0. Unlike previous optical skyrmions, these SLSP skyrmions are intrinsic eigenmodes of the metasurface. This offers a key advantage: they don't require complex external excitation and are easily excited by simple sources. Furthermore, the skyrmions topology is robust to shape deformations as Figure 2. Owing to spatial‐spiral guiding mechanism, skyrmions multi‐resonance maintain stability even when metasurface undergoes continuous deformation into various geometries.

## 3 Capacitance Loading for Enhanced Bending Stability in SLSP Skyrmions

**FIGURE 3:**
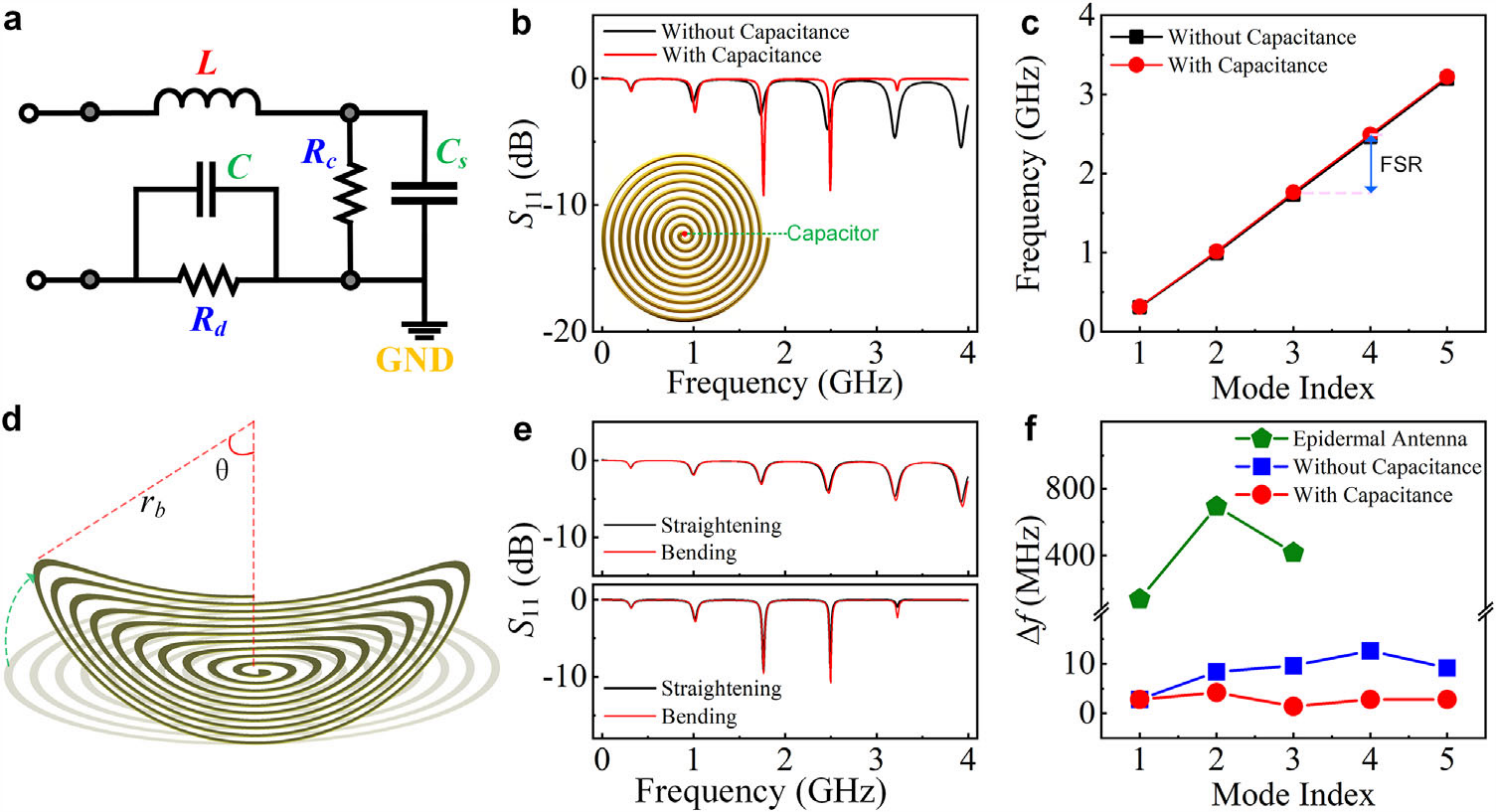
(a) Equivalent *LC* circuit model. (b) *S*‐parameters showing blueshift under capacitor loading. (c) The linear relationship between resonant mode index and frequency, maintained as a linear function (confirming preserved FSR). (d) Schematic drawing of SLSP skyrmions bending. (e) Effect of capacitive loading on the bending‐induced frequency shift: multi‐resonance response of the SLSP skyrmions without (top) and with (bottom) capacitance loading at a bending angle of θ = 30°, corresponding to *r_b_* = 27/*π* mm. (f) The relationship between mode index and the bending‐induced frequency shift Δ*f* for the common antenna and the SLSP skyrmions, with and without capacitive loading, at a bending angle of θ = 30° (corresponding to *r_b_* = 27/*π* mm).

For the topological robustness of SLSP skyrmions, it will be obtained the applications in deformation artifact‐free sensing. In this paper, the capacitance loading on space‐coiling meta‐structure is introduced to enhance the bending stability of the sensor. A quasi‐static equivalent circuit model for capacitance loading on skyrmions was established as Figure 3a. *L* corresponds to the magnetic energy stored in the structure, and *C* is determined by the distributed capacitance between neighboring turns of the spiral. *R_c_* and *R_d_* represent the conductor loss and dielectric loss, respectively. While *C_s_* represents a capacitance connected in series at the ground terminal. Herein, the equivalent circuit model for space‐coiling meta‐structure was established, extending the established expressions for square planar structures [27, 28]. The calculations for *L* and *C* were provided below

(3) L=μ02πlNrlnllNrNr2(d−a)+12

(4) $$C=C_{0}\frac{\pi N_{r}^{3}}{8\left(N_{r}^{2}+1\right)}\left[\frac{\pi N_{r}d}{2}\left(N_{r}-1\right)-\frac{N_{r}^{2}-1}{2}d\right]$$

where *C*_0_ denotes the perunit‐length capacitance between two parallel trips, whose is related to the dielectric substrate, as follows

(5) $$C_{0}=\varepsilon_{0}\varepsilon_{r,sub}\frac{\kappa \left(1-\sqrt{k^{2}}\right)}{\kappa \left(k\right)}$$

(6) $$\varepsilon_{r,sub}\left(\varepsilon_{r},t,d\right)=1+\frac{2}{\pi }\mathrm{arctg}\left[\frac{t}{2\pi d}\right]\left(\varepsilon_{r}-1\right)$$

where *ε*_0_, κ( · )and *t* denote the vacuum permittivity, the complete elliptic integral of the first kind and the thickness of the dielectric substrate, respectively, k=a/a(2d−a)(2d−a). It should be noted that *ε_r,sub_* converges to unity when the dielectric substrate thickness diminishes.

The impedance *Z* of the equivalent circuit of the original SLSP skyrmions is as follows

(7) $$Z=R_{c}+{\left(\frac{1}{j\omega L}+j\omega C+\frac{1}{R_{d}}\right)}^{-1}$$

When the capacitance C_s_ is connected in series at the ground terminal, the impedance of the entire circuit will become the original impedance connected in series with the impedance of *C_s_*, due to that *C_s_* is connected in series with the ground terminal, when viewed from the input port, *C_s_* is connected in series with the original circuit. To simplify the derivation and ignore the losses (*R_c_* and *R_d_*), thus the total impedance *Z_total_* is expressed as

(8) $$Z_{total}=\frac{j\omega L}{1-\omega^{2}LC}+\frac{1}{j\omega C_{s}}$$

When in a resonant state, the imaginary part of the total impedance is 0, as follows

(9) $$Im\left(Z_{total}\right)=\frac{j\omega L}{1-\omega^{2}LC}-\frac{1}{j\omega C_{s}}=0$$

Hence, an analytical expression for the fundamental resonant frequency is given by

(10) $$f_{0}=\frac{1}{2\pi \sqrt{L(C+C_{s})}}$$

Therefore, microwave resonators are fundamentally equivalent to *LC* resonant circuits, whether it is a lumped parameter or a distributed parameter model. Introducing series/parallel capacitive elements adds an extra capacitance to this equivalent circuit. Capacitors, as energy storage components, exhibit frequency‐dependent reactance, in which the expression is as follows

(11) $$X_{C}=\frac{1}{2\pi fC}$$

When the SLSP skyrmions is loaded with a capacitance bending‐induced reactance variations become negligible relative to the loaded capacitance value. Therefore, the dominant factor governing resonant frequency shift is the capacitance value of the loaded element.

Herein, a capacitor was connected in series between the skyrmions and ground plane. The series‐capacitance alters the coupling condition between the resonator and the external circuit. In the vicinity of the resonance, the series capacitor introduces an additional negative reactance (‐*jX_C_*). To compensate for this reactance and achieve resonance, the equivalent inductive reactance (*X_L_* = 2*πfL*) of the resonator itself must increase. Therefore, the necessitates an elevation in the resonant frequency. Full‐wave electromagnetic simulations compared the *S*‐parameters of both unloaded‐ and loaded‐capacitance SLSP skyrmions as shown in Figure 3b, in which Rogers RO4003C was as the dielectric substrate with the dimensions of20mm × 20mm × 1mm. It can be seen that capacitance loading induces a blueshift across all resonant modes, with larger shifts observed for higher‐order modes. The results consistent with the *X_L_* = 2*πfL* relationship. Furthermore, capacitance loading enhances the quality factor (Q‐factor). The Q‐factor is defined as Q=f0/f0ΔfBWΔfBW, in which *f*_0_ is the resonance frequency and *f_BW_* is the bandwidth where *S*_11_ drops 3 dB lower than the top. The largest Q‐factor among the first five modes increases from 22 to 163 compared with unloaded and loaded capacitor. Figure 3c showed the mode number vs. frequency relationship for both configurations, revealing overlapping linear trends. It was demonstrated that SLSP skyrmions supported near‐equidistant multi‐resonant responses in both cases with and without capacitance. Meanwhile, we performed a statistical analysis of the free spectral range (FSR) for the first five resonant modes under two configurations (with and without capacitive loading). The mean FSR (µ), standard deviation (σ), and coefficient of variation (CV = σ/µ × 100%) were calculated. The results show µ = 0.722 GHz and σ = 23.5 MHz (CV = 3.26%) without capacitance, and µ = 0.727 GHz and σ = 17.7 MHz (CV = 2.44%) with capacitance. This extremely small relative variation quantitatively confirms the preservation of equally spaced resonances.

The impact of the bending angle (θ) of the resonator on its resonant frequency was further investigated via simulation, as shown in Figure 3d. Unless otherwise specified, the bent state corresponds to θ = 30°, *r_b_* = 27/*π* mm. To underscore the topological protection mechanism of skyrmions, its performance was compared with that of a common antenna of identical size (11 mm × 13.1 mm) under bending conditions; the comparative changes in the magnitude of *S*_11_ and the resonant frequency were presented in Figure 3e and Figure S2. Figure S2 showed the significant degradation of the common antenna's response with bending [13], where a substantial shift in both the resonant frequency and the *S*_11_ magnitude is observed, culminating in a maximum frequency shift of 693 MHz. Due to the distinct resonance mechanisms, the common antenna relies on conventional distributed resonance strongly dependent on its effective electrical length, which is directly perturbed under bending. However, the SLSP skyrmion modes arise from a space‐coiling, topologically protected magnetic resonance mechanism that is less sensitive to geometric deformation. In contrast, Figure 3e showed the spectrum comparison of SLSP skyrmions under both straight and bent states, with and without capacitance loading. The topological protection mechanism resulted in remarkable stability: the bent SLSP skyrmions exhibited an average frequency shift of merely 8.5 MHz across their resonant modes. Moreover, the incorporation of capacitive loading further suppressed bending‐induced frequency drift, reducing the average shift for all modes to 2.8 MHz, as shown in Figure 3f. Therefore, the capacitively loaded SLSP skyrmions mitigated the impact of bending on the frequency shift by approximately 250 times compared to the common antenna.

**FIGURE 4:**
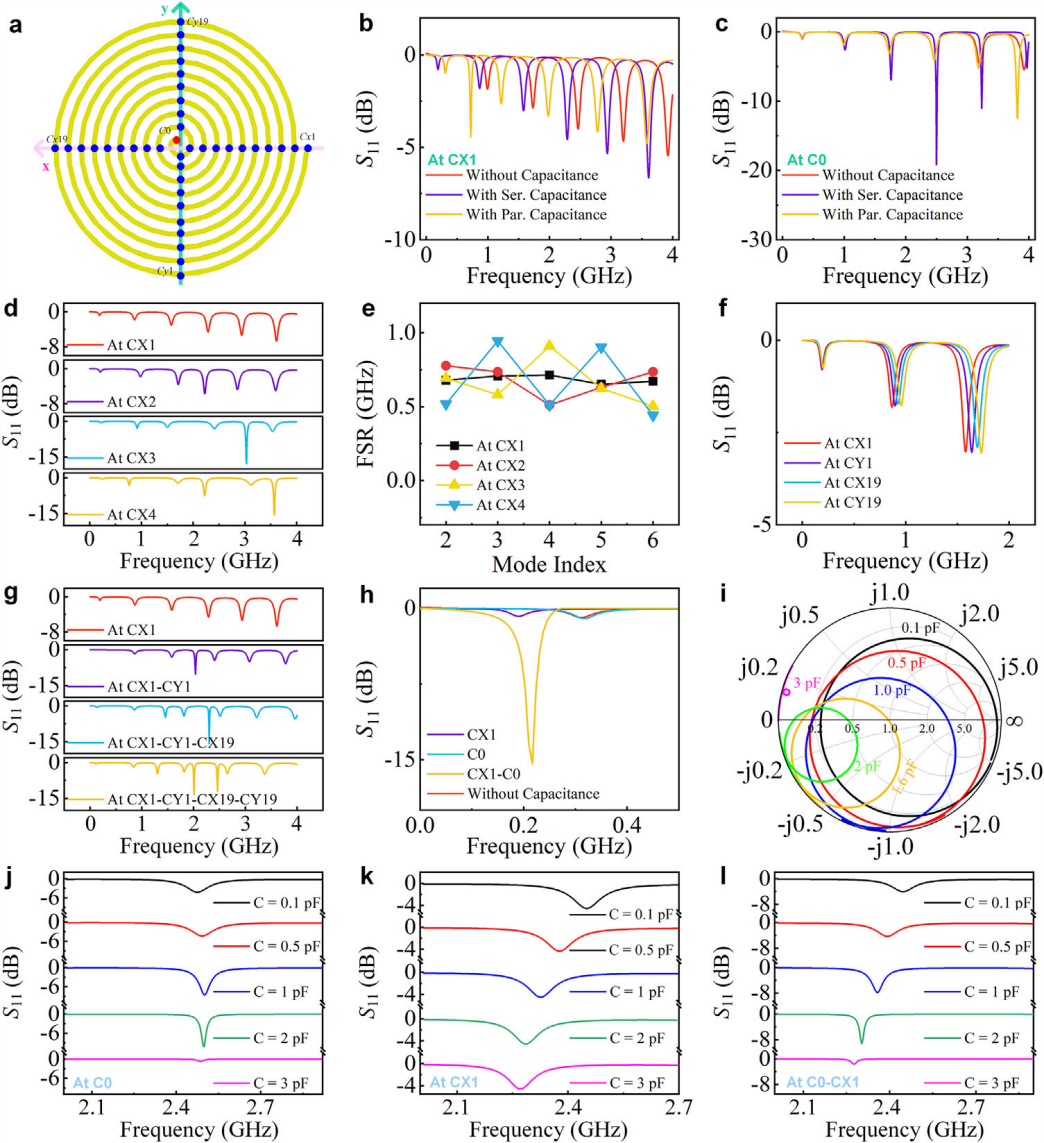
Tunable response of SLSP skyrmions through capacitor loading. (a) Schematic of capacitor loading positions (marked as blue dots) on the SLSP skyrmion structure. *S*‐parameters of SLSP skyrmions under different loading conditions: (b) at position CX1 and (c) at position C0, comparing capacitor‐free, series‐capacitor, and parallel‐capacitor loading. (d) *S*‐parameters for series‐capacitor loading at positions CX1, CX2, CX3, and CX4. (e) Relationship between FSR and mode index for series‐capacitor loading at CX1, CX2, CX3, and CX4. (f) *S*‐parameters for series‐capacitor loading at CX1, CY1, CX19, and CY19. (g) *S*‐parameters for series‐capacitor loading at single and combined positions: CX1, CX1‐CY1, CX1‐CY1‐CX19, and CX1‐CY1‐CX19‐CY19. (h) Comparison of *S*_11_ spectra: without capacitor, with series‐capacitor at CX1, at C0, and at both CX1 and C0. (i) Smith chart representation of *S*_11_ variation with series‐capacitance. *S*_11_ spectra as a function of series‐capacitance at: (j) C0, (k) CX1, and (l) both C0 and CX1.

To analyze the impact of capacitance element position on the electromagnetic properties of SLSP Skyrmions, we defined loading positions along the *x*‐ and *y*‐axes. Figure 4a depicted the capacitive loading points (blue markers), arranged in a grid pattern with positions labeled CX1 to CX19 on the *x*‐axis, CY1 to CY19 on the y‐axis, and a central point labeled C0. To clarify the experimental implementation of capacitive loading, the physical configurations of the series and parallel capacitors are illustrated in Figure S3. We performed electromagnetic simulations incorporating 2 pF parasitic capacitances connected in series/parallel at both terminals (CX1 and C0) of the SLSP skyrmion, respectively. Observing the results reveals that capacitor loading at the skyrmion's leading end (C0) produces a pronounced alteration in resonant frequency, meanwhile, when a capacitor is loaded at the end (edge position) CX1, the resonant

Depth and Q value are greatly increased. Second, when capacitors are connected in parallel, the resonant frequency undergoes a redshift, and the resonant frequency undergoes a blueshift for series. This is because connecting capacitors in parallel increases the total capacitance. It follows from Equation (11) that an increase in capacitance reduces the capacitive reactance at a given frequency. Furthermore, the fundamental resonance condition for an *LC* circuit, expressed in Equation (10), dictates an inverse proportionality between the resonant frequency and $\sqrt{C}$. Thus, the increase in capacitance is the direct cause of the observed decrease in resonant frequency.

To further analyze the impact of loading capacitors at different positions on the SLSP skyrmions spectrum, we take the four positions along the X‐direction (CX1, CX2, CX3, CX4) shown in Figure 4a as the region of interests (RoI). The simulation results for loading series‐ and parallel‐capacitance at these four positions are shown in Figure 4d and Figure S4a, respectively. Meanwhile, according to the value of FSR as Figure 4e and Figure S4b, it can be observed that only when the capacitor is loaded at the edge position CX1 does the SLSP maintain its equidistant resonance. In contrast, loading capacitors at the other three positions disrupts the skyrmion's equidistant resonance pattern.

The non‐uniform frequency perturbation causes originally equidistant modes to shift to varying degrees. Simultaneously, the lifting of mode degeneracy splits the originally single resonance peak into two. These two effects work in concert to completely destroy the equidistant characteristic of the resonant frequencies. Consequently, the observed resonance spectrum becomes complex, with peak positions no longer uniformly distributed. To provide more direct evidence of symmetry breaking, we added a comparison of the simulated magnetic‐field distributions for the sixth resonant mode with capacitive loading at CX1 and CX4 (Figure S5). With edge loading at CX1, the field pattern remains nearly concentric and symmetric, which is consistent with preserved equidistant resonances. In contrast, loading at CX4 causes field distortion and asymmetry, indicating symmetry breaking and non‐uniform modal perturbation, which leads to resonance splitting and degradation of the equal‐spacing (FSR) characteristic.

This characteristic is sometimes exploited for actively controlling skyrmions modes, such as selectively exciting specific modes or generating topologically protected boundary states. However, this inevitably comes at the cost of sacrificing the original equidistant property. Therefore, to preserve this characteristic, capacitors should be loaded at the periphery or the beginning of the spiral structure.

Additionally, to analyze the influence of different peripheral positions on the resonant frequency, four positions (CX1, CY1, CX19, CY19) were selected for series‐ and parallel‐capacitance loading analysis, as shown in Figure 4f and Figure S6. The simulation results reveal that, moving clockwise from the end, the resonant frequency undergoes a blueshift while still maintaining the equidistant resonance characteristic.

For progressively loading series‐ and parallel‐capacitance clockwise from the CX1 to CY19, however, the characteristic of SLSP skyrmions was broken, as shown in Figure 4g and Figure S7. It is undesirable for this work. To preserve the symmetry of SLSP skyrmions and avoid lifting mode degeneracy, this work focuses solely on the two positions at the space‐coiling structure's beginning and end. Four schemes were analyzed by electromagnetic simulation software, including unloaded capacitance, loaded capacitance at the C0, loaded capacitance at the CX1, and simultaneous capacitance loading at both. The results demonstrated that loaded capacitance at the C0 caused a redshift in the resonant frequency, while loaded capacitance at the CX1 induces a slight blueshift, as shown in Figure 4h. However, when capacitors are loaded simultaneously at both ends, the resonant frequency undergoes a redshift through iterative interaction of the two modes, while concurrently yielding a multiplicative enhancement in resonance depth and Q‐factor. Therefore, the scheme of loaded capacitances at both the CO and CX1 of the SLSP skyrmions adopted. This approach significantly enhances resonance depth, Q‐factor, and the resonator's bending stability while maintaining equidistant resonant characteristics.

Finally, the influence of varying capacitance values on the SLSP skyrmions spectrum was analyzed and visualized on the Smith Chart. The Smith Chart provides an intuitive visualization of the impedance matching state, with the center point (50 Ω) indicating perfect matching (no reflection). A trajectory closer to the center signifies lower reflection and higher excitation efficiency. Figure 4i showed the trajectories of the reflection coefficient for the SLSP skyrmions across the frequency range under different capacitance loading. As the value of capacitance increases, the resonant structure transitions from undercoupling to overcoupling. When C = 2 pF, the trajectory approaches closest to the center point, indicating critical coupling. Figure 4j–l and Figure S8 showed the impact of capacitance variation on the resonant frequency for three configurations, which were series‐ and parallel‐capacitance at position C0, at position CX1 and simultaneously at both C0 and CX1, respectively. Meanwhile, Table S1 gave the summary for capacitor‐loading configurations and their impacts. Figure 4j demonstrates that when loading capacitance at C0, the resonance depth changes with capacitance value. As capacitance increases from 0.1 to 2 pF, the resonance depth improves from ‐4.225 to‐10.123 dB. Beyond 2 pF, the resonance depth decreases with further capacitance increase, indicating an overcoupling regime. Therefore, the highest resonance depth of ‐10.123 dB is achieved at 2 pF in Figure 4j, corresponding to a total Q‐factor of 178.5. For series‐capacitance loaded at CX1, the resonant frequency exhibits a progressive blueshift with increasing capacitance. Figure 4k showed that a variation in load capacitance from 0.1 to 2 pF induces a corresponding change in the compressed resonant wavelength from λ/13.6 to λ/14.6 (λ is the free‐space wavelength). The blueshift becomes marginal beyond 2 pF, with the resonant wavelength reaching only λ/14.7 at 3 pF. When series capacitors are simultaneously loaded at both the head and tail positions (C0 and CX1) of the skyrmion structure, the resonant characteristics represent a superposition of the previous two configurations, as presented in Figure 4l. Therefore, optimal resonance depth and frequency blueshift are simultaneously achieved at a capacitance value of 2 pF. For the resonance mode of C = 2 pF in Figure 4l, the resonance diameter is compacted into λ/14.5, while the resonance depth is reached to ‐9.253 dB with a total Q‐factor of 164.29. Subsequently, when the SLSP skyrmions is employed as a sensor, a capacitance value of 2 pF was selected for analysis.

**FIGURE 5:**
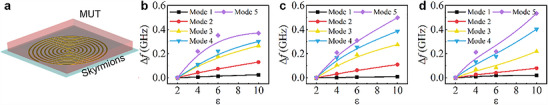
(a) Schematic of SLSP skyrmions loaded material under test (MUT). The measured frequency shifts of the first five modes based on three configurations, including (b) capacitor‐free loading, (b) series‐capacitor loading and (c) parallel‐capacitor loading.

Above on analysis, it is concluded that the SLSP magnetic skyrmions modes were excited to enhance topological robustness of the sensor, while bending stability along the longitudinal direction was improved by capacitance loading. Therefore, the introduced capacitance‐loading SLSP skyrmions as a wearable sensor achieved higher detection stability and motion‐free sensing. Three sensing configurations were comparatively analyzed through simulations: (i) capacitance‐free loading, (ii) series‐capacitance loading, and (iii) parallel‐capacitance loading, shown in Figure 5. Resonator's response was ‌utilized to characterize the different material under test (MUT). To facilitate comparative analysis, the first five resonant modes were selected from the magnetic skyrmions, and the corresponding results were displayed in Figure 5b–d. The analysis reveals that higher‐order modes exhibit increased sensitivity but greater susceptibility to noise, accompanied by reduced correlation between permittivity variations and sensor response. Comparative assessment of the three configurations demonstrates that capacitance loading enhances both detection sensitivity and response correlation. This improvement stems from the increased Q‐factor of the *S*‐parameters, which subsequently elevates sensitivity and detection resolution. When the dielectric constant of the MUT increased from 2 to 10, the resonance frequency shifts of Mode 5 for both series‐ and parallel‐capacitance configurations were approximately 0.5 GHz, meanwhile, the correlation coefficients of 0.98 and 0.97 were achieved, respectively. Therefore, the optimal design selected in this work employs a pair of series capacitances loading at the head and tail of the space‐coiling meta‐structure, realizing a microwave sensor with high stability and sensitivity.

## 4 Experimental Validation of the SLSP Skyrmions Sensing Performance

**FIGURE 6:**
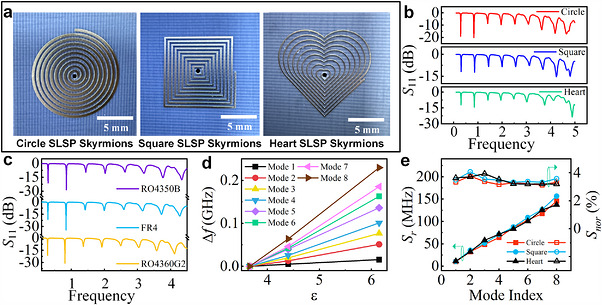
(a) Fabricated circle‐, square‐, and heart‐shaped SLSP skyrmions on Rogers 5880. (b) Comparison of S‐parameters for the three sensor geometries. (c) S‐parameters measured for the circle‐ shaped SLSP skyrmions with three types of MUTs. (d) Corresponding Δ*f* of the first eight resonant modes. (e) Calculated detection resolution and normalized sensitivity for three geometries SLSP skyrmions.

To validate the high topological robustness of the proposed SLSP skyrmions and their feasibility for characterizing the permittivity of low‐loss solid materials, the proposed SLSP skyrmion sensor was fabricated and measured. Figure 6a showed three SLSP skyrmions with distinct topological configurations, including circular, square and heart‐shaped, fabricated on a Rogers 5880 dielectric substrate with the dimensions of20mm × 20mm × 1mm, while maintaining identical effective waveguide lengths (*l* = 128 mm). On the unloaded MUT condition, these three sensors exhibited nearly identical reflection characteristics, as shown in Figure 6b. It could be seen that all three configurations were achieved nine near‐equidistant resonances within the 5 GHz bandwidth. These resonances represent the first nine resonant modes of SLSP skyrmions, with each mode maintaining consistent resonant frequencies across the different shapes, thereby demonstrating the high topological robustness of the SLSP skyrmions. The frequency intervals between adjacent resonant modes are not strictly equidistant due to the influence of scattering losses, lossy conductors, and the dielectric substrate. In this paper, to enhance detection precision and achieve broadband multimode detecting, the first eight resonances were employed for the measurements.

To characterize its sensing performance, the introduced SLSP skyrmions was tested with three different low‐loss MUTs (Rogers RO4350B, FR4, Rogers 4360G2). The key properties of MUTs, including permittivity (*ε_r_*), loss tangent (tan *δ*) and thickness (*h_mut_*), are listed in Table 1. By sequentially placing each MUT sample of identical dimensions onto the proposed SLSP skyrmions sensor, the corresponding *S*_11_ parameters were measured, as shown in Figure 6c. It is evident that as the MUT is changed from Rogers 4350B to Rogers 4360G2, the increasing permittivity of the MUT induces a redshift in all eight resonant modes. Consequently, for each MUT, its permittivity (*ε_mut_*) is detected by acquiring the frequency shift (Δ*f*) of each resonant peak. A second‐order curve fitting method can then be adopted to establish a mathematical sensing model for each resonant mode, as follows

(12) $$\varepsilon_{mut}=a\times \Delta f^{2}+b\times \Delta f+c$$

where Δ*f* denotes the shift in resonant frequency between the loaded MUT and reference states, while a, b, and c represent fitting coefficients. Therefore, a set of 8 mathematical sensing models can be constructed, one for each resonant mode. The corresponding measurement data and fitted curves are illustrated in Figure 6d. Each mode yields an independent permittivity estimate using its calibrated fitting curve; the final output can be obtained by weighted averaging across modes and/or discarding outliers, thereby improving robustness against noise, coupling fluctuations, or imperfect MUT placement.

**TABLE 1:** Key parameters of the three tested materials (MUTs).

Material	*ε_r,mut_*	tan*δ*	*h_mut_* (mm)
Rogers RO4350B	3.66	0.0031	0.762
FR4	4.4	0.02	0.5
Rogers RO4360G2	6.15	0.0035	0.635

To quantify the performance of SLSP skyrmions sensor, two evaluation metrics commonly used in the sensor field are adopted, such as detection resolution *S_r_* and normal sensitivity *S_nor_*. The *S_r_* is given by the ratio of Δ*f* to the variation in refractive index Δ*n*, in which $n=\sqrt{\varepsilon_{n}}$ [29], expressed as follows

(13) $$S_{r}=\frac{\Delta f}{\Delta n}$$

This indicates that the sensor can accurately detect the minimum change in material permittivity. Figure 6d shows the resonance shift Δ*f* of the eight resonant modes of SLSP skyrmions corresponding to the MUT dielectric constant from small to large.

The *S_nor_* is another core sensor metric, evaluated using the formula:

(14) $$S_{nor}=\frac{\Delta f}{f_{re}\cdot \Delta n}$$

where *f_re_* denotes the resonant frequency of reference state. Based on Figure 6d, the evaluation metrics (*S_r_* and *S_nor_*) of each resonant mode of SLSP skyrmions were calculated according to Equations (13) and (14), as shown in Figure 6e. It could be observed that the *S_r_* of broadband multi‐resonant skyrmions sensor were enhanced with the increase of resonant modes and the *S_r_* of 156.5 MHz was achieved for the eighth mode, and the high normalized sensitivity (*S_nor_* > 3.1%) was achieved and exhibited high stability. Furthermore, the *S_r_* and *S_nor_* values for the three differently shaped sensors closely matched, demonstrating strong consistency in sensing performance despite variations in geometry. To ensure comparability, the same experimental setup and MUTs were maintained uniformly across all three sensor geometries. Therefore, the results demonstrated that when the effective waveguide length is kept identical, these sensors maintain topologically robust sensing characteristics even during continuous geometric deformation.

**FIGURE 7:**
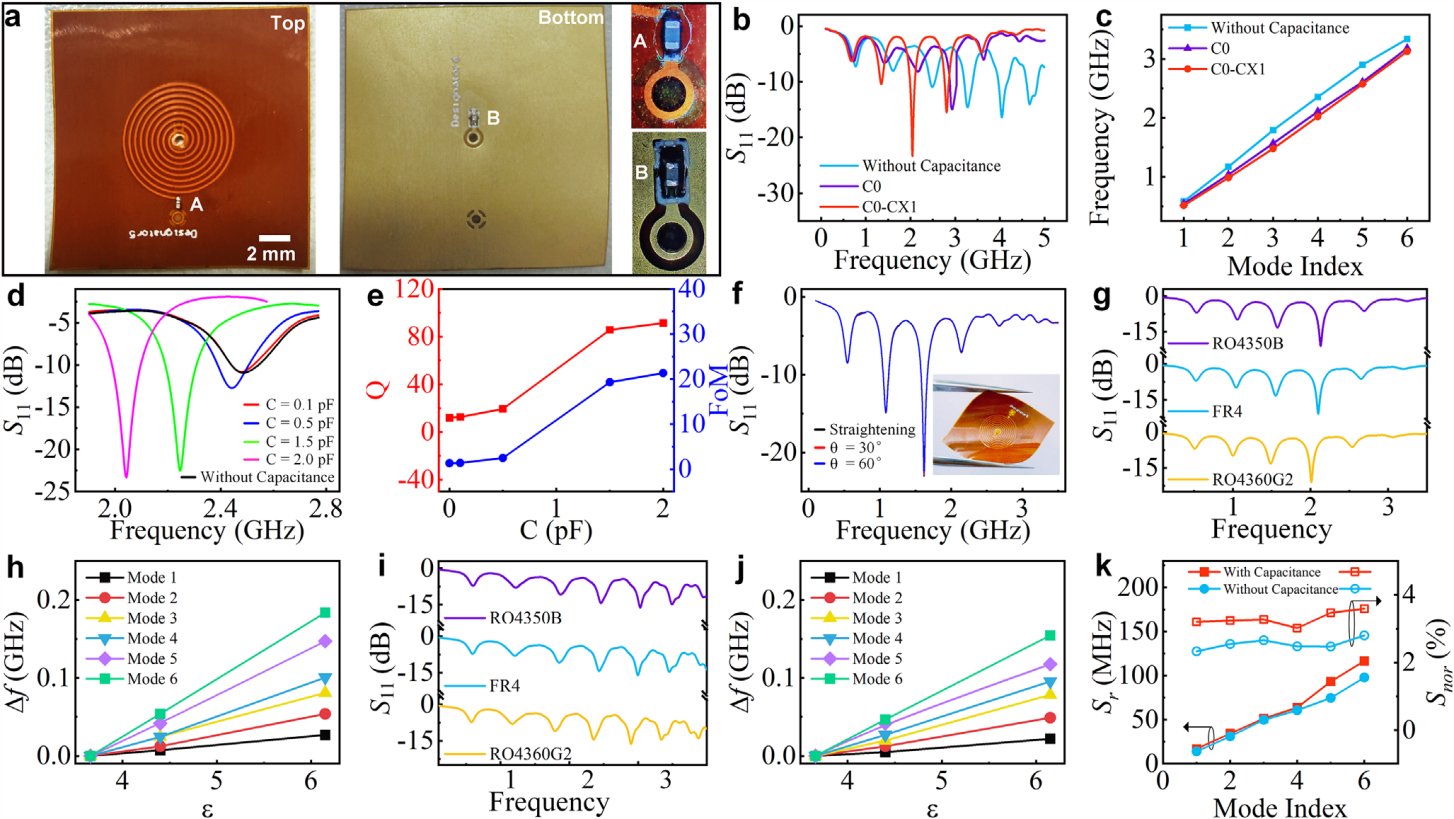
Flexible SLSP skyrmion sensor with capacitors loading. (a) Fabricated circle‐, square‐, and heart‐shaped sensors on a flexible polyimide (PI) substrate; inset shows loaded capacitors. (b) Measured *S*_11_ spectra without a capacitor, with a capacitor at C0, and with capacitors at both C0 and CX1. (c) Resonance frequency vs. mode index derived from (b). (d) *S*_11_ spectra as a function of capacitance. (e) performance metrics (Q factor and FoM), varying with the capacitance. (f) Comparison of S‐parameters of SLSP skyrmions under straightening, bending of 30°, and bending of 60°. (g) S‐parameters for three MUT samples measured with capacitor‐loaded sensor and (h) corresponding frequency shifts of the first six modes. (i) *S*‐parameters for MUT samples without a capacitor and (j) associated frequency shifts. (k) Resolution and normalized sensitivity comparison for sensors with and without capacitor loading.

To facilitate wearable integration, the designed SLSP skyrmions structure was printed onto a flexible polyimide (PI) substrate with the dimensions of20mm × 20mm × 0.2mm, as shown in Figure 7a. In accordance with the capacitive loading approach outlined in Section 3, two surface‐mount capacitors (0201 package) were implemented at the lower terminals of the resonator, as shown in Figure 7a, to enhance the sensor's stability under vertical bending. Figure 7b showed the measured *S*_11_ parameters for three configurations: unloaded capacitor, loaded capacitor only at position C0, and simultaneous loaded capacitors at both ends (C0‐CX1). The results were shown that capacitance loading increased the maximum Q‐factor from 49.35 to 90.24. Furthermore, capacitance loading induced a redshift in the resonant frequency, consistent with the conclusions drawn in Section 3. All three configurations maintained near‐equidistant resonance, as illustrated in Figure 7c, with the configuration featuring loading at both ends (C0‐CX1) exhibiting a lower FSR in *S*_11_ compared to the other two schemes.

Additionally, the impact of loading capacitors with different capacitance values on the reflection coefficient of the SLSP Skyrmions resonator was analyzed. Optimal performance in resonator excitation necessitates concurrently high excitation efficiency and Q‐factor, since these parameters together reflect the system's interaction with its environment [30]. To enable quantitative assessment of excitation quality, a Figure of Merit (FoM) is proposed, expressed as

(15) FoM=Q×δI

The term *δI* (unit of dB) corresponds to the resonance depth of *S*_11_, which serves as a measure of excitation efficiency. Figure 7d shows the reflection coefficients corresponding to different capacitor values. It can be observed that increasing the capacitance value decreases the resonant frequency while simultaneously yielding a higher reflection peak and an increased *Q*‐factor. This increasing trend tends to saturate when the capacitance reaches 2 pF, as depicted in Figure 7e.

To validate the bending stability of the proposed SLSP skyrmions structure, the skyrmion structure was conformally mounted on semi‐cylindrical foam models with bending angle of *θ* = 30° and 60°, as shown in Figure S9. The resonator responses under straightening conditions (no bending), bending of 30° and bending of 60° were

collected, as depicted in Figure 7f (Enlarged views of resonance modes were shown in Figure S10). It can be observed that the responses under these three states exhibit near‐perfect overlap. Therefore, this demonstrates that the proposed capacitance loading SLSP skyrmions possess exceptionally high bending stability as a microwave sensor. The obtained results are highly encouraging. Applications in electronic skin, and ultra‐compact antenna are now facilitated by the emergence of magnetic SLSP skyrmions, which offer a path to durable and highly integrated plasmonic systems.

The impact of capacitance loading on the sensing performance of the SLSP skyrmions was further analyzed. RO4350B, FR4, and RO4360G2 materials were separately detected using both the capacitance loading SLSP skyrmions and the capacitance‐free loading SLSP skyrmions, as shown in Figure 7g–j. The first six resonance modes were selected as RoI, shown in Figure 7h,j. For both sensor types, the first six resonance modes exhibited a positive correlation between the mode index and detection resolution. Furthermore, capacitance loading enhanced the *S_r_* of sensor, as shown in Figure 7k. This enhancement results from the higher resonance mode Q‐factor achieved through capacitor loading. For a comprehensive comparison, the *S_nor_* results are presented in Figure 7k. It can be observed that capacitor loading yields superior sensing performance for all six resonance modes compared to unloaded capacitor SLSP skyrmions. Therefore, the proposed SLSP skyrmions sensor offers a more compact footprint, enhanced sensing performance, and more robust topological characteristics.

## 5 Conclusion

In this work, we propose an ultra‐compact multi frequency sensing strategy for SLSP magnetic skyrmions based on multi‐capacitance loading to characterize the dielectric constant in broadband. In addition, it can strongly limit the wavelength within the deep subwavelength range and suppress radiation loss. Owing to its strongly localized fields and topological stability, the magnetic skyrmions enable highly sensitive and precise sensing, even under continuous shape deformation. Meanwhile, the SLSP skyrmions were loaded with multiple capacitors, and their resonant frequency achieved extremely high bending stability. By changing the capacitance of the load capacitor, SLSP skyrmions with deep subwavelength, high Q value, and high resonance depth can be obtained. Finally, SLSP skyrmions were prepared separately on Rogers 5880 and PI substrates for detecting MUTs with different dielectric constants. Prove that the sensitivity of SLSP skyrmions sensors can be improved through capacitive loading. SLSP magnetic skyrmions have exciting features such as high integration, ultra‐compact, topological stability, and multi‐mode sensing capabilities, and are expected to demonstrate enormous potential in a range of potential applications. And the topological robustness and vertical bending stability of continuous deformation have opened up possibilities for its application in wearable devices.

## Conflicts of Interest

The authors declare no conflicts of interest.

## Supporting information




**Supporting File**: advs75071‐sup‐0001‐SuppMat.pdf.

## Data Availability

The data that support the findings of this study are available upon reasonable request from the authors.
