# Supplementary material for: Reconfigurable Spoof Plasmonic Skyrmion Electronics for Deformation‐Invariant Multimode Sensing
Source: Adv Sci (Weinh). 2026 Apr 8;13(34):e75071. doi: 10.1002/advs.75071 (PMC13285175; doi:10.1002/advs.75071)
Supplement: Supplementary file 1 — Supporting File: advs75071‐sup‐0001‐SuppMat.pdf. [file ADVS-13-e75071-s001.pdf]

# Supplementary Materials for

## Reconfigurable Spoof Plasmonic Skyrmion Electronics for Deformation-Invariant Multimode Sensing

**This file includes:**

Supplementary Figures S1 to S10
Supplementary Table S1
Supplementary Notes 1 to 2

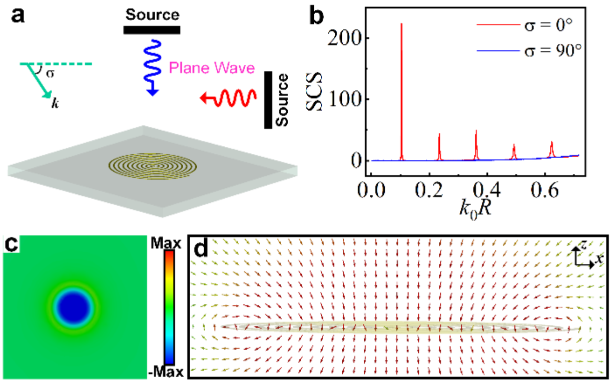


**Fig. S1 Excitation and characterization of resonant modes. For all the modes in the near-equidistant spectrum, the results were manifested purely magnetic modes.** (a) Simulation setup illustrating horizontal (along y-axis) and vertical (along x-axis) wave incidence directions. (b) Corresponding scattering cross-section (SCS) spectra. (c) Vertical magnetic field (Hz) distribution 0.5 mm above the structure at the first resonance frequency for horizontal incidence. (d) Magnetic field lines within the y=0 plane at the first resonance frequency for horizontal incidence.

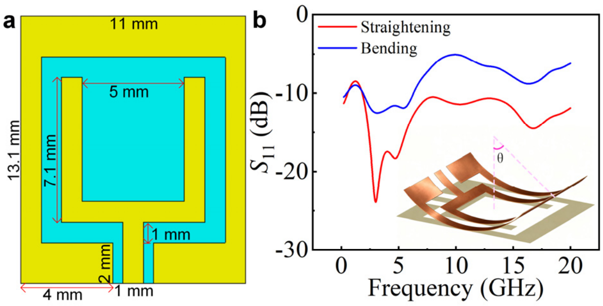


**Fig. S2** (a) The geometry and dimensions of the epidermal antenna, (b) Comparison of S11 in the epidermal antenna under straight and bent conditions [13].

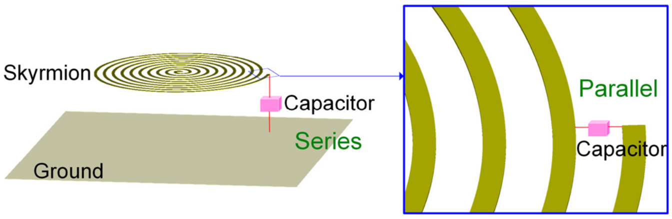


**Fig.S3** Schematic illustration of the physical implementation of capacitor loading. Left: series capacitor connected between the skyrmion resonator node and the ground plane. Right: parallel capacitor bridging the gap between adjacent metallic rings (zoomed-in view).

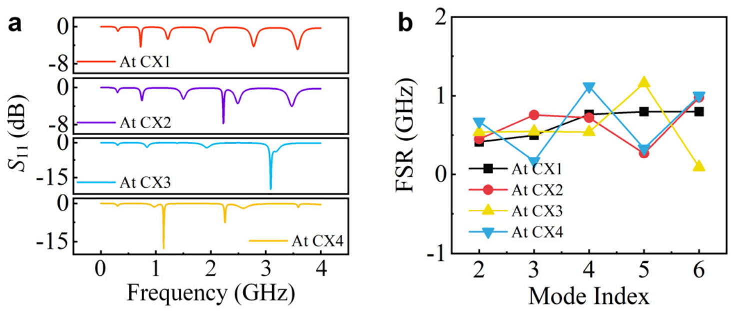


**Fig.S4** (a) *S*-parameters for parallel-capacitor loading at positions CX1, CX2, CX3, and CX4. (b) Relationship between FSR and mode index for parallel-capacitor loading at CX1, CX2, CX3, and CX4.

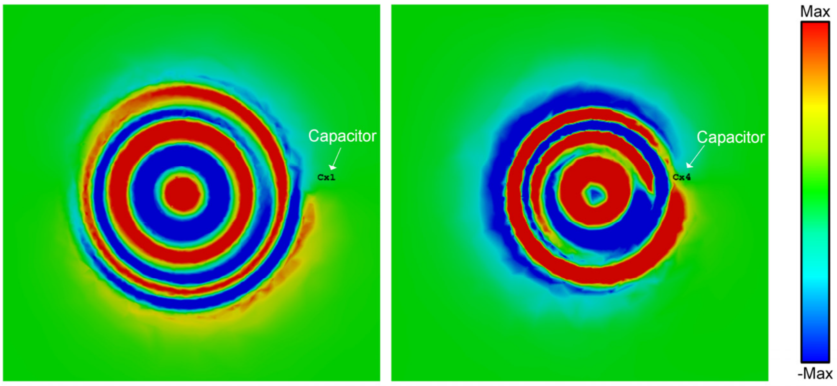


**Fig. S5** Magnetic field distributions of the sixth resonant mode with capacitors loaded at CX1 and CX4. Edge loading at CX1 preserves the rotational symmetry of the skyrmion mode, while interior loading at CX4 breaks symmetry and induces spatial field distortion, leading to non-uniform modal perturbation.

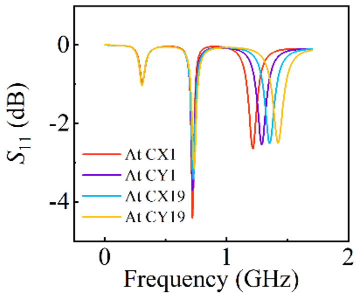


**Fig.S6** *S*-parameters for parallel-capacitor loading at CX1, CY1, CX19, and CY19.

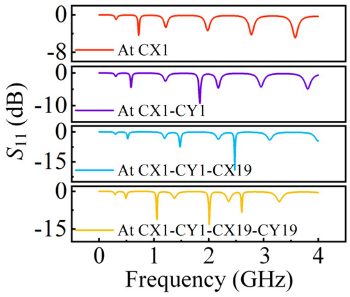


**Fig.S7** *S*-parameters for parallel-capacitor loading at single and combined positions: CX1, CX1-CY1, CX1-CY1-CX19, and CX1-CY1-CX19-CY19.

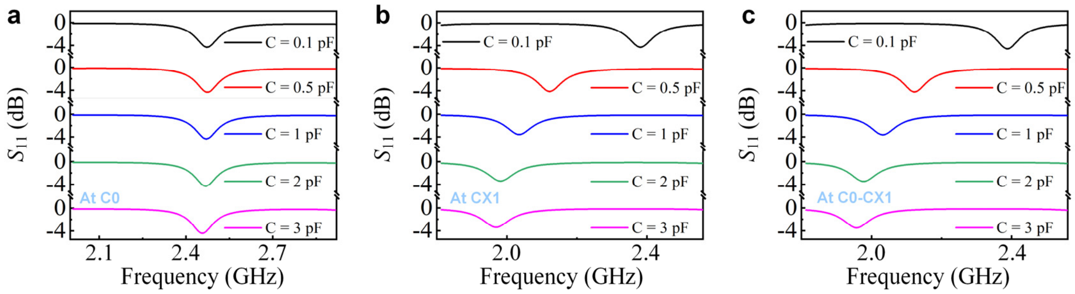


**Fig.S8** $S_{11}$ spectra as a function of parallel-capacitance at: (a) C0, (b) CX1, and (c) both C0 and CX1.

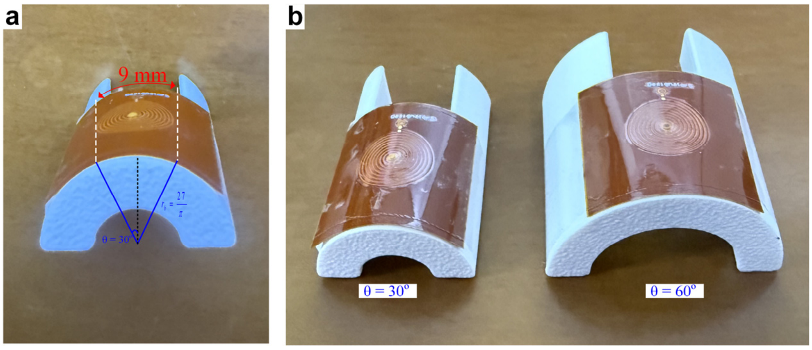


**Fig.S9** (a) the relationship illustration between bending angle and radius, and (b) Illustration of the skyrmion conformally bent at the bending of 30° and 60°.

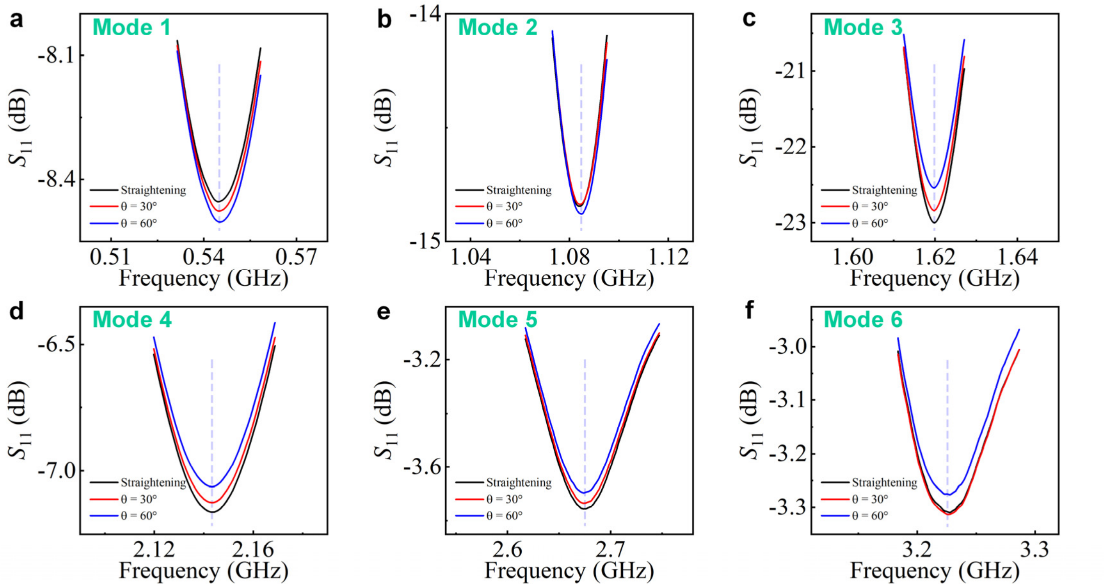


**Fig. S10** Comparison of all the modes in the near-equidistant spectrum under straightening, bending of 30°, and bending of 60°. All resonant modes perfectly overlap in the three bending states.

**Table S1** Summary table for capacitor-loading configurations and their impacts

| | Capacitor loading | Capacitance (pF) | $f_0$ (GHz) | Q | Resonance depth (dB) |
|---|---|---|---|---|---|
| Series | C0 | 0.1 | 2.475 | 15.372 | -4.225 |
| | | 0.5 | 2.492 | 16.394 | -4.318 |
| | | 1 | 2.501 | 71.457 | -8.517 |
| | | 2 | 2.499 | 178.5 | -10.123 |
| | | 3 | 2.487 | \ | -0.666 |
| | CX1 | 0.1 | 2.450 | 18.015 | -4.519 |
| | | 0.5 | 2.378 | 16.986 | -4.324 |
| | | 1 | 2.326 | 18.315 | -4.500 |
| | | 2 | 2.287 | 18.901 | -4.556 |
| | | 3 | 2.270 | 19.076 | -4.572 |
| | C0−CX1 | 0.1 | 2.445 | 14.298 | -4.064 |
| | | 0.5 | 2.392 | 16.611 | -4.326 |
| | | 1 | 2.357 | 51.239 | -7.773 |
| | | **2** | **2.303** | **164.29** | **-9.253** |
| | | 3 | 2.275 | \ | -1.647 |
| Parallel | C0 | 0.1 | 2.470 | 16.25 | -4.310 |
| | | 0.5 | 2.470 | 16.25 | -4.333 |
| | | 1 | 2.470 | 16.578 | -4.373 |
| | | 2 | 2.470 | 16.358 | -4.296 |
| | | 3 | 2.457 | 17.55 | -4.429 |
| | CX1 | 0.1 | 2.382 | 17.014 | -4.288 |
| | | 0.5 | 2.121 | 14.235 | -4.123 |
| | | 1 | 2.035 | 19.019 | -3.591 |
| | | 2 | 1.981 | 9.955 | -3.391 |
| | | 3 | 1.969 | 15.146 | -3.402 |
| | C0−CX1 | 0.1 | 2.387 | 19.25 | -4.582 |
| | | 0.5 | 2.123 | 15.384 | -4.217 |
| | | 1 | 2.032 | 9.407 | -3.642 |
| | | 2 | 1.976 | 7.689 | -3.485 |
| | | 3 | 1.958 | 7.770 | -3.496 |

## Supplementary Notes

### Supplementary Note 1. Model for the Space-coiling Meta-structure

The designed subwavelength meta-surface consists of arranged metallic curves. This structure forms metallic resonant cavities. Under incident electromagnetic waves in the microwave frequency range, it generates Fabry–Pérot (FP)-like resonances, where structural resonance replaces plasmonic resonance. Consequently, the surface plasmon resonances of the meta-surface also exhibit characteristics of traditional resonators. The subwavelength nature of the meta-surface enables extremely high resonant frequencies and quality factors within relatively small structural dimensions. Furthermore, the coherent interference arising from the repeated reflection of surface plasmons within the resonant structure can lead to the enhancement of localized fields on the meta-surface.

In this model, the space-coiling structure is equivalent to a metal-insulator-metal perfect electric conductor (PEC) waveguide that is closed on one side and open on the other. The space-coiling meta-structure comprises a series of semicircles with progressively increasing radii. The radii of the $i^{\mathrm{th}}$ ($i$ = 1, 2, ..., $n$) upper semicircle and the $i^{\mathrm{th}}$ lower semicircle are given by Eq. (1) and Eq. (2), respectively.

$$r_1^i = (2i-1)\frac{d}{2} \tag{1}$$

$$r_2^i = id \tag{2}$$

The arrangement of the metallic lines forms air-filled dielectric regions, effectively creating an air waveguide with an effective length $l$. The total effective waveguide length is as follows

$$l = \sum_{i=1}^{N_r} \pi\left(r_1^i + r_2^i\right) = \pi(N_r^2 d + \frac{1}{2}N_r d) \tag{3}$$

Where $N_r$ denotes the number of turns of the spiral. Under near-field excitation by an incident transverse magnetic (TM) polarized plane wave, SLSP bounce between the air waveguides, collectively oscillating to form FP-like resonances. The resonant frequency is determined by the effective length $l$ of the air waveguide within the meta-surface. According to the FP resonance condition

$$\frac{\lambda_m}{2} + (m-1)\lambda_m = 2l,\ m = 1,2,3,\cdots,M \tag{4}$$

Therefore, the resonant wavelength for the $m^{\mathrm{th}}$ mode of the SLSP is

$$\lambda_m = \frac{4l}{2m-1},\ m = 1,2,3,\cdots,M \tag{5}$$

The resonance condition in terms of normalized frequency $k_0R$ is described as

$$k_0 R = (m + \frac{1}{2})\pi\frac{R}{S} \tag{6}$$

where $R = N_r \times d$. According to Eq. (3) and Eq. (6), it can be calculated

$$k_0 R = \frac{2m+1}{2N_r+1} \tag{7}$$

The spacing between adjacent modes, or the free spectral range (FSR) yields, is given by

$$\text{FSR} = \frac{2}{2N_r+1} \tag{8}$$

Based on the above equations, the resonance positions for each mode can be approximately predicted, with the precise resonance peaks subsequently identified through full-wave finite element simulations.

**Supplementary Note 2. Purely Magnetic SLSP Skyrmions**

To analyze the excitation conditions of the equidistant resonances of the SLSP skyrmions, full-wave simulations were performed for cases of horizontally incident (along the $y$-axis) and vertically incident (along the x-axis) electromagnetic waves, as shown in Fig. S1a. The scattering cross-section (SCS) spectra produced by incident waves from these two directions differ significantly, as shown in Fig. S1b. For horizontal incidence (red curve), multiple equidistant resonance peaks are observed in the SCS, whereas no resonances are generated for vertical incidence (blue curve). To identify the resonant mode of the first peak under horizontal incidence, the corresponding magnetic field and magnetic field lines were simulated and calculated, as shown in Fig. S1c-d. Figure S1c shows the distribution of the vertical magnetic field component ($H_z$) at a height of 0.5 mm above the structure. It can be observed that at resonance, the magnetic field is strongest at the center of the structure, and the phase distribution is consistent across the structure surface, indicating resonance along the z-direction. Furthermore, Fig. S1d presents the magnetic field strength and field line distribution within the cross-section at y=0. The magnetic field lines are shown to form closed loops, passing through the center of the structure. This pattern is consistent with the field line distribution of a magnetic dipole oriented along the $z$-axis.

Therefore, when the size of the SLSP structure is relatively large, the electric field phase varies at different positions on the resonator surface under illumination by a horizontally incident electromagnetic wave, due to a phase delay effect. This leads to the excitation of higher-order resonant modes. When the electromagnetic wave is incident vertically, the entire surface of the resonant structure lies within the same phase front of the plane wave, preventing the excitation of higher-order resonant modes. This characteristic is very similar to the resonant behavior of traditional localized surface plasmons (LSPs).
